# Study on the Therapeutic Effects of Bisdemethoxycurcumin on a Cerebral Amyloid Angiopathy Mouse Model Established via Chronic Treatment With Five Vascular Risk Factors

**DOI:** 10.1002/brb3.70245

**Published:** 2025-01-19

**Authors:** Shudong Lin, Guanghua Zhu, Juan Xie, Xuanwei Wen, Limin Deng, Sijing Li, Guozhi Liu, Feiyan Wang, Shuangxi Chen, Zijian Xiao

**Affiliations:** ^1^ Department of Neurology, The First Affiliated Hospital, Multi‐Omics Research Center for Brain Disorders, Hengyang Medical School University Of South China Hengyang Hunan China; ^2^ Clinical Research Center for Immune‐Related Encephalopathy In Hunan Province, Hengyang Medical School University Of South China Hengyang Hunan China; ^3^ Department of Emergency, The First Affiliated Hospital, Hengyang Medical School University of South China Hengyang Hunan People's Republic of China

**Keywords:** cerebral amyloid angiopathy (CAA), curcumin, learning and memory, necroptosis, neuroinflammation

## Abstract

**Background and Purpose:**

Cerebral amyloid angiopathy (CAA) is recognized as a major contributor to progressive cognitive decline and cerebral hemorrhages in the elderly population. Currently, there is a global shortage of safe and effective treatments for this condition. Bisdemethoxycurcumin (BDMC) has been demonstrated to exhibit pharmacological effects with anti‐Aβ toxicity properties. Thus, the present study mainly focused on the potential therapeutic effects of BDMC on CAA.

**Method:**

The 30 male C57BL/6 mice were subjected to chronic treatment with five vascular risk factors (lipopolysaccharide, social stress, streptozotocin, high‐cholesterol diet, and copper‐containing drinking water) for 35 weeks to establish a CAA mouse model. Of these, 15 CAA mice received oral administration of BDMC (50 mg/kg) for two consecutive weeks as an intervention, while the remaining 15 CAA mice received an equal volume of physiological saline by gavage. The study observed the levels of Aβ40 and proinflammatory factors in brain tissue and plasma, Aβ deposition in cerebral blood vessels, microbleeds in brain tissue, expression of proteins related to the cGAS/STING signaling pathway in brain tissue, as well as the contents of p‐RIPK‐1, p‐RIPK‐3, p‐MLKL, neuronal morphology, and learning and memory abilities in mice.

**Result:**

The therapeutic administration of BDMC demonstrates a pronounced efficacy in alleviating Aβ burden and cerebral microbleeding in CAA mice, concurrently enhancing learning and memory capabilities. Interestingly, BDMC may inhibits neuroinflammatory responses by reducing the expression of cGAS/STING signaling pathway proteins and suppresses necroptosis.

**Conclusion:**

Our research findings demonstrate that BDMC exerts therapeutic effects in a mouse model of CAA established through chronic treatment involving five vascular risk factors.

## Graphical abstract

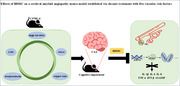

## 1 Introduction

Cerebral amyloid angiopathy (CAA) is a cerebral microvascular disease characterized by the deposition of insoluble amyloid‐beta (Aβ) in the cerebral cortex and leptomeningeal blood vessel walls, which results in increased fragility of blood vessels and localized neurotoxicity (Koemans et al. 2023). Clinical manifestations primarily encompass recurrent or multiple cerebral lobar hemorrhages, cognitive impairment, dementia, and psychiatric symptoms (Charidimou et al. 2017). Currently, there is a significant challenge in finding effective treatments for spontaneous intracerebral hemorrhage (ICH) and the cognitive dysfunction associated with CAA. Research has consistently indicated that the abnormal deposition of Aβ in the walls of cerebral blood vessels plays a pivotal role in the pathogenesis of CAA. This deposition disrupts endothelial cell tight junctions and triggers perivascular inflammatory responses, ultimately leading to neurodegeneration, neuroinflammation, and neurovascular cell damage in the context of CAA (Greenberg et al. 2019; Sweeney et al. 2019). Therefore, the search for a safe and effective clinical drug targeting Aβ remains crucial, as it has the potential to provide significant benefits to individuals with CAA.

**FIGURE 1:**
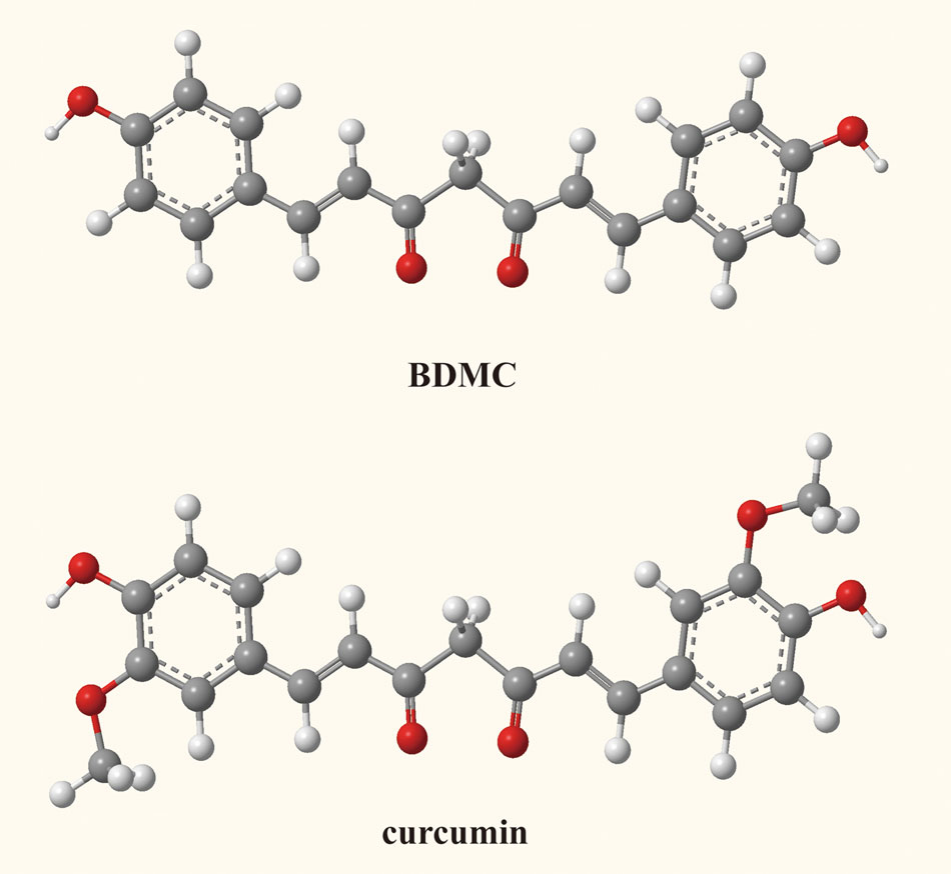
The structure of curcumin and BDMC.

Research suggests that curcumin can impact the synthesis and elongation of Aβ, degrade Aβ fibers, and thereby reduce Aβ‐induced neurotoxicity and provide neuroprotection (Veldman et al. 2016; Gagliardi et al. 2022). However, curcumin has low water solubility, minimal polarity, is susceptible to degradation in alkaline environments, and faces challenges in crossing the blood‐brain barrier (Young, Rai, and Nitin 2020). Additionally, rapid metabolism to tetrahydrocurcumin in rat, mouse, and human liver cells further reduces its effectiveness (Sood et al. 2023; Wu et al. 2023). Bisdemethoxycurcumin (BDMC), a derivative of curcumin, eliminates the 3‐methoxy group from both sides of the phenyl rings while retaining the hydroxyl group at the 4‐position (Figure 1). BDMC exhibits higher polarity, water solubility, and metabolic stability compared to its parent compound, showcasing more significant anti‐Aβ toxicity (Ramezani, Hatamipour, and Sahebkar 2017; Xie et al. 2023). Preliminary experiments by our research team have demonstrated that BDMC significantly improves learning and memory functions in transgenic APP/PS1 mice, indicating its anti‐Aβ toxicity effects (Xu et al. 2020a; Xu et al. 2020b). While there are no report on BDMC treating CAA, considering its pharmacological actions and the pathological mechanisms of CAA, we speculate that BDMC may be an effective approach for ameliorating CAA.

Here, we establish a CAA mouse model to explore the impact of BDMC on CAA. Our findings show that BDMC administration reduces amyloid protein burden and CAA pathology, suppresses neuroinflammatory responses by decreasing cGAS/STING signaling pathway protein expression, inhibits necrotic apoptosis, and improves cognitive abilities associated with CAA. These discoveries suggest that BDMC may hold promise as an effective therapeutic agent for CAA.

## 2 Materials and Methods

### 2.1 Animals

Due to the potential influence of hormones on drug efficacy, male mice were exclusively utilized in this study. Male C57BL/6 mice (5‐month‐old, 25–35 g) were obtained from Changsha Tianqin Biotechnology Co., Ltd. The mice were housed in the Specific Pathogen‐Free (SPF) animal facility at University of South China, with conditions maintained at approximately 21±1°C, 40–55% relative humidity, and a 12/12‐h light/dark cycle. Food and water were available ad libitum. All experimental procedures received ethical approval from the Laboratory Animal Ethics Committee of the First Affiliated Hospital of University of South China (approval No. 2022LL0523001) and were designed and reported according to the Animal Research: Reporting of In Vivo Experiments (ARRIVE) guidelines (Boutron et al. 2020).

### 2.2 CAA Model

After 1 week of acclimatization, 5‐month‐old C57/BL6 mice underwent a specific experimental protocol as follows: In the first week, intraperitoneal injections of lipopolysaccharide (LPS) were administered; in the third week, intraperitoneal injections of streptozotocin were given; during the fifth week, cage changes were accompanied by social stress. Additionally, the mice were fed a high‐cholesterol diet and provided with copper‐containing drinking water for 5 weeks, followed by 2 weeks of normal diet and water. Each cycle lasted 7 weeks, and this cycle was repeated five times, totaling 35 weeks. The modeling process concluded when the animals reached 14 months of age. The high‐cholesterol diet contains 397 g/kg corn starch, 200 g/kg casein, 132 g/kg maltodextrin, 100 g/kg sucrose, 70 g/kg soybean oil, 50 g/kg fiber, 35 g/kg mineral mixture, 3 g/kg L‐cysteine, 2.5 g/kg choline chloride, 0.014 g/kg butylhydroxytoluol, 10 g/kg vitamin mix, 1 g/kg chocolate aroma, 0.002 g/kg folic acid, and additional 50 g/kg cholesterol (Collaborative Pharmaceutical Bioengineering Co., Ltd; Jiangsu Province, China).

**FIGURE 2:**
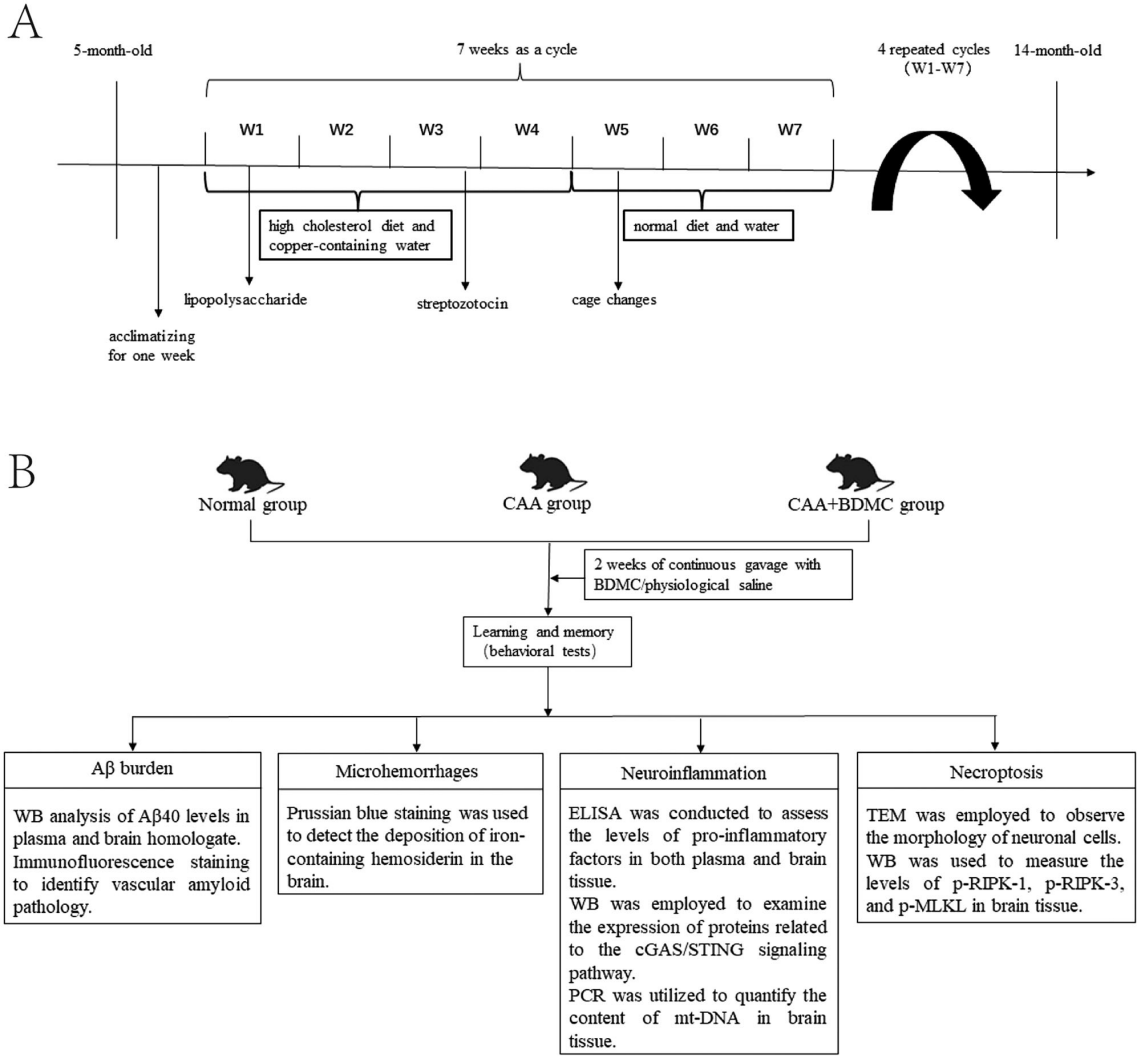
Study design. Representative images of CAA mice modeling. Representative images of experimental process.

Copper‐containing drinking water referred to standard drinking water supplemented with 1 mg/L of anhydrous copper sulfate. For intraperitoneal LPS injections, the dosage was 5 mg/kg in the first cycle and 1.25 mg/kg in subsequent cycles. Streptozotocin was initially administered at a dose of 15 mg/kg in the first cycle, with a subsequent increase of 5 mg/kg in each cycle, reaching a maximum dose of 35 mg/kg (Figure 2A).

### 2.3 Preparation of BDMC Solution

BDMC (consisting of more than 80% curcumin and more than 94% curcuminoid content) (Cat# 33171‐05‐0, Sigma‐Aldrich, St. Louis, MO, USA) was dissolved in phosphate‐buffered saline (PBS) containing 0.5% dimethyl sulfoxide, as described in a previous study (Parlar and Arslan 2019).

### 2.4 Treatment Groups

The 45 male C57BL/6 mice were randomly divided into three groups: the Normal group, CAA group, and CAA+BDMC group (*n* = 15 each). The CAA group and CAA+BDMC group underwent the CAA model establishment using the methods described earlier. The Normal group was fed a regular diet and provided with normal drinking water for 35 weeks. During the modeling process involving intraperitoneal injections of lipopolysaccharide and streptozotocin, the Normal group received simultaneous intraperitoneal injections of physiological saline. After 35 weeks, the Normal group and CAA group received 2 weeks of continuous gavage with physiological saline, while the CAA+BDMC group received 2 weeks of continuous gavage with BDMC (50 mg/kg). Subsequently, all mice underwent behavioral tests. After completing the tests, the mice were euthanized, and blood plasma and brain tissue homogenates were collected for enzyme‐linked immunosorbent assay (ELISA). Brain tissues from the mice were also subjected to immunofluorescence staining, Prussian blue staining, transmission electron microscopy, and protein immunoblot analysis (Figure 2B).

### 2.5 Behavior Tests

#### 2.5.1 Y‐Maze

The Y‐maze consists of three arms, designated as the novel arm, the start arm, and the other arm. These arms are labeled A, B, and C, respectively. Mice are placed in the overlapping region of the three arms, and their activities in the maze are recorded for a duration of 5 min. Correct alternation is defined as the mouse consecutively entering three different arms (A‐B‐C, A‐C‐B, B‐C‐A, B‐A‐C, C‐A‐B, C‐B‐A), while incorrect alternation occurs if, within three consecutive entries, the mouse enters the same arm twice (A‐B‐A, A‐C‐A, B‐C‐B, B‐A‐B, C‐A‐C, C‐B‐C, A‐B‐B, A‐C‐C, B‐C‐B, B‐A‐B, C‐A‐C, C‐B‐C, A‐A‐C, A‐A‐B, B‐B‐A, B‐B‐C, C‐C‐B, C‐C‐A, C‐C‐C, A‐A‐A, B‐B‐B). Following each individual mouse experiment, the Y‐maze is cleaned with high‐concentration alcohol to prevent residual odors from interfering with test results. The number of correct alternations within a 5‐min period is recorded for each mouse. The spontaneous alternation rate is calculated as the total number of correct alternations divided by the total number of arm entries across all three arms, multiplied by 100%. The spontaneous alternation rate is directly proportional to the spatial recognition ability of the mice.

#### 2.5.2 Novel Object Recognition (NOR)

A novel object recognition task was conducted to examine nonspatial learning and memory (Leger et al. 2013). The experiment consisted of two trials, each lasting 5 min, with an inter‐trial interval of 15 min. In the first trial, two of the same objects were put in the open field arena. In the second, one object was replaced by a novel object, while the other object remained the same and in the same location. The time spent exploring both objects was counted, and novel object recognition was examined through evaluating the discrimination index (DI). DI = (time with novel object‐time with familiar object)/(time with novel object‐time with familiar object).

#### 2.5.3 Morris Water Maze

To evaluate the effect of BDMC on the spatial learning and memory capacity of mice, the Morris water maze test was performed as described previously (Vorhees and Williams 2006). Briefly, the maze (Cat# XR‐XM101, Shanghai Xinruan Information, Technology, Co., Ltd., Shanghai, China), a plastic, circular pool 1.2 m in diameter and filled with water at 22°C to a depth of 31 cm, was divided into four quadrants (A, B, C, and D). A transparent platform was submerged 1 cm under the surface of the water in the C quadrant. This test comprised three phases: the acquisition trial, the probe trial, and the visible platform test. During the acquisition trial, mice were placed in the water in one of the four quadrants and allowed to swim freely for up to 60 s to find the platform. The escape latency was defined as the time needed for the mouse to find the transparent platform. If the mouse did not find the platform within 60 s, the escape latency was recorded as 60 s, and the investigator then guided the mouse to the platform, where it was allowed to remain for 15 s. This trial was performed once a day for 4 consecutive days. On the fifth day, the transparent platform was removed. The mice were placed in the water in the opposite quadrant and allowed to swim freely for 60 s. The degree of memory consolidation was indicated by the number of times that the mouse crossed the area were the platform had been and the time spent in the target quadrant. On the sixth day, a visible platform was installed in the pool to test the visual and motor function of each mouse and exclude the effect of the possible deficits in vision or motor processing in the prior experiment. The platform was located 2 cm above the surface of the water in the target quadrant. The time needed to reach the platform and the average swimming speed were recorded.

### 2.6 Tissue Preparation

Following the conclusion of behavioral experiments, mice were anesthetized via intraperitoneal injection of hydrate 2.5% Avertin based on their body weights. Subsequently, three mice from each group underwent cardiac perfusion, and the entire brains were then processed for paraffin embedding and sectioning. The remaining mice in each group had fresh brain tissues collected for subsequent immunoblot analysis, PCR, and enzyme‐linked immunosorbent assay (ELISA). Additionally, four mice from each group were selected to collect eyeball blood specimens for ELISA.

### 2.7 Western Blot Assay

Wash fresh brain tissue with cold PBS 2–3 times, and then homogenize it thoroughly in 10 times the tissue volume of cold RIPA lysis buffer. Samples were kept on ice for 30 min, vortexed for 5–10 min, centrifuged at maximum speed for 10 min, and the supernatant was collected to determine protein levels using the Bicinchoninic Acid Assay. Lysates were subsequently separated by SDS‐PAGE gels and transferred topolyvinylidenedifluoridemembranesfollowingstandardtechniques.Membranes were blocked with 5% nonfat dried milk in Tris‐buffered saline containing 0.1% Tween‐20. Blots were incubated overnight with the following primary antibodies: anti‐STING (anti‐rabbit, 1:1000), anti‐TBK1 (anti‐rabbit, 1:1000), anti‐p‐TBK1 (anti‐rabbit, 1:1000), anti‐IRF3 (anti‐rabbit, 1:1000), anti‐p‐IRF3 (anti‐rabbit, 1:1000), anti‐p65‐NF‐κB (anti‐rabbit, 1:1000), anti‐p‐p65‐NF‐κB (anti‐rabbit, 1:1000), anti‐p‐PIPK1 (anti‐rabbit, 1:1000), anti‐p‐PIPK3 (anti‐rabbit, 1:1000), anti‐p‐MLKL (anti‐rabbit, 1:1000), and anti‐glyceraldehyde 3‐phosphate dehydrogenase (GAPDH) (anti‐mouse, 1:5000) as loading control. Band intensity was quantified using the ImageJ analysis software.

### 2.8 Immunofluorescence Staining

Mice were anesthetized by intraperitoneal (i.p.) injection of 2.5% Avertin and immediately perfused using 4% paraformaldehyde in PBS. Brains were then excised, postfixed in 4% paraformaldehyde at 4°C overnight, and incubated in 30% sucrose at 4°C until equilibrium. Subsequently, the brain tissues were embedded in O.C.T. compound blocks. Sequential 10‐µm coronal sections were obtained using a cryostat for further analysis. The frozen brain sections were washed using PBS. Subsequently, brain sections were incubated with 10% normal donkey serum (Solarbio) for 1 h at room temperature for blocking the nonspecific binding. Following this, sections were incubated with primary antibodies overnight at 4°C. After PBS washing, sections underwent a subsequent incubation with the corresponding fluorescent‐labeled secondary antibody for 1 h at room temperature in the dark. Next, high‐temperature antigen retrieval was performed, reopening antigen binding sites, followed by the addition of another set of primary antibodies and their corresponding secondary antibodies. The primary antibodies used included anti‐CD‐31 (1:100; Abcam, Cat. No. ab182981) and anti‐beta amyloid 1–40 (Aβ40) (1:200; PTG, Cat. No. 25524‐1‐AP). Secondary antibodies comprised Donkey anti‐rabbit Alexa Fluor 647 (1:400; Abcam, Cat. No. ab150075) and Donkey anti‐rabbit Alexa Fluor 488 (Abcam, Cat. No. ab150073). Finally, the brain sections were counterstained with 4’,6‐diamidino‐2‐phenylindole (DAPI) (Beyotime) for nuclear visualization. Immunofluorescence staining results were captured using a fluorescent microscope.

### 2.9 Cytokine Analysis

Proinflammatory cytokines (IL‐1β, IL‐6, IL‐8, TNF‐α, INF‐β, and cGAMP) levels were determined in protein extracts by ELISA following the manufacturer's procedure.

### 2.10 qRT‐PCR Analysis

For qRT‐PCR analysis, mouse brain tissue DNA was extracted using a yeast genomic DNA extraction kit of Baiao Mobo Technology (Beijing, China) following the supplier's instructions. (Invitrogen, USA). Transcript expression levels were examined using on a 7300 Real‐Time PCR System (Applied Biosystems) using QuantiTect SYBR Green I (Qiagen, USA) as a fluorescent dye. The QuantiTectOneStep RT‐PCR Kit was used for subsequent gene‐specific amplification. The primer sequences for the RT‐PCR experiments were supplied by Generay Biotechnology (Shanghai, China), and the specific sequences were as follows: mtDNA (forward 5 ′‐GCCCCCGATATGGCGTTT‐3′; reverse 5′‐GTTCAACCTGTTCCTGCTCC‐3′), and β‐actin (forward 5′‐ATGGATGACGATATCGCTG‐3′, reverse 5′‐GTTGGTAACAATGCCATGTTC‐3′). Relative gene expression levels were analyzed using the comparative CT method (ΔΔCT). The expression level of target genes was standardized to the housekeeping gene GAPDH.

### 2.11 Prussian Blue Staining

The tissue sections were sequentially placed into xylene I (20 min)–xylene II (20 min)–absolute ethanol I (5 min)–absolute ethanol II (5 min)–75% ethanol (5 min), followed by rinsing with tap water and distilled water (3 times each). A Prussian blue staining solution was prepared by mixing potassium ferrocyanide solution and hydrochloric acid solution in equal proportions. The sections were immersed in the staining solution for 1 h, followed by rinsing with distilled water (2 times). Nuclear fast red staining was performed for 1–5 min, followed by rinsing under running water. Subsequently, the sections were sequentially immersed in absolute ethanol I (5 min)–absolute ethanol II (5 min)–absolute ethanol III (5 min)–xylene I (5 min)–xylene II (5 min) for dehydration and transparency. The sections were then mounted with neutral mounting medium. Microscopic examination was conducted, and images were captured and analyzed.

### 2.12 Transmission Electron Microscope (TEM) Analysis

Brain tissues were harvested and cut in 1 mm^3^, prefixed in 2% glutaraldehyde, and fixed in 1% osmium tetroxide. Then, samples were dehydrated in ethanol using 3% uranyl acetate, embedded in the epoxy resin and propylene oxide overnight, and polymerised. After sectioning into 70‐nm‐thick sections and staining with lead citrate, the sections were detected by H‐7650 transmission electron microscope (Hitachi, Japan) at 300 kV.

### 2.13 Statistical Analysis

Data represented as mean ± standard error of the mean (SEM) unless otherwise indicated. All analysis were repeated independently with similar results at least three times. Statistical analysis was conducted using GraphPad Prism 10.0. Differences between two groups were analyzed by Student's *t* test. One‐way analysis of variance (ANOVA) with Tukey's post hoc tests were performed for comparisons between multiple groups. *p* Value < 0.05 was considered statistically significant.

## 3 Results

### 3.1 BDMC Enhances Learning and Memory in CAA Mice

**FIGURE 3:**
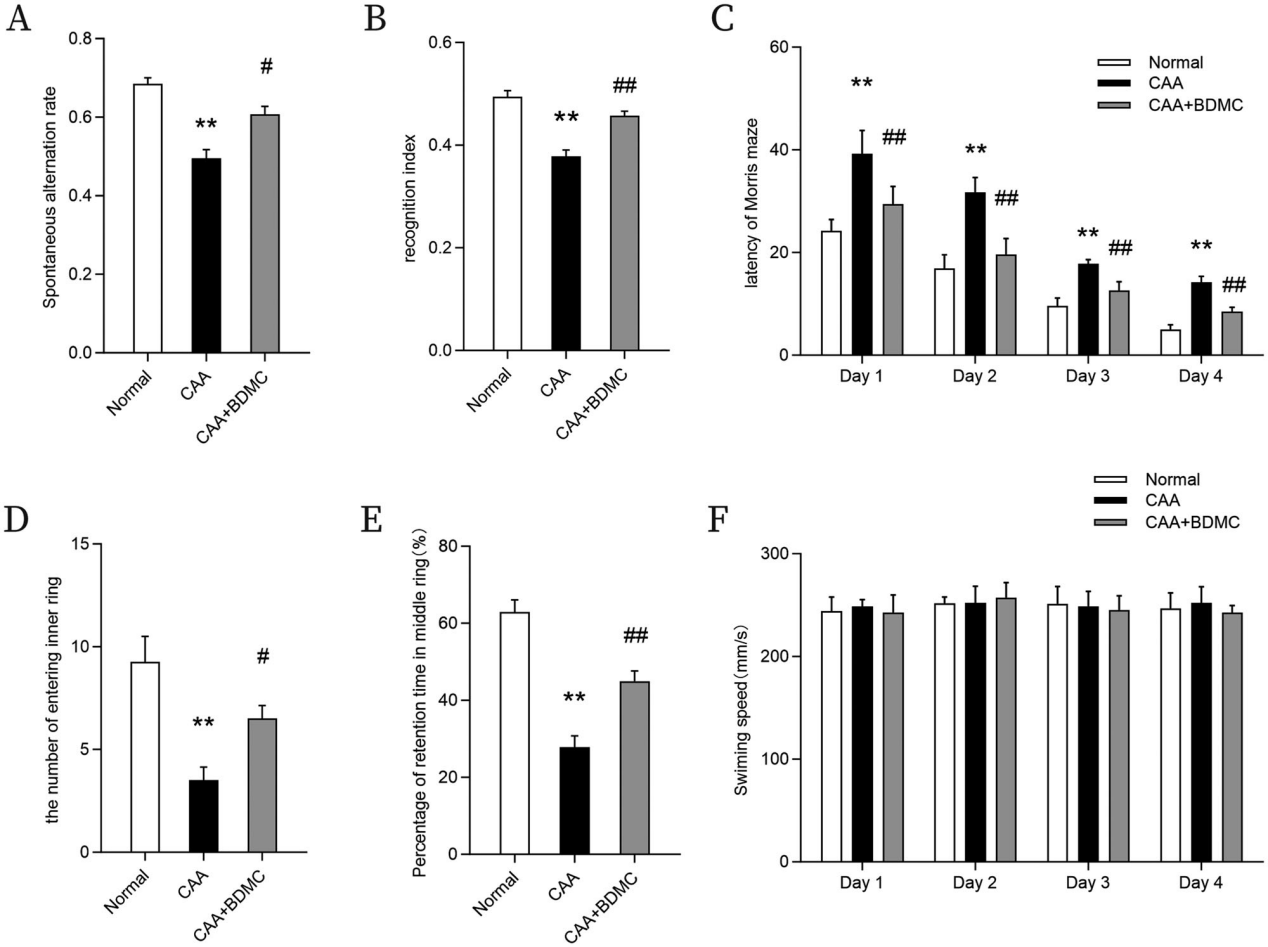
BDMC enhances learning and memory in CAA mice. Spontaneous alternation rate of mice in Y‐maze. The recognition index of mice in novel object recognition experiments. (C–F) Morris water maze: (C) escape latency, (D) the number of entering inner ring, (E) percentage of retention time in middle ring, and (F) swimming speed. Data are shown as the mean ± SEM. Compared to the normal group: *p < 0.05, **p < 0.01; Compared to the CAA group: #*p* < 0.05, ##*p* <0.01.

Initially, we assessed the effect of BDMC on the learning and memory of CAA mice. We found that normal mice of similar age and gender outperformed CAA mice in Y‐maze, novel object recognition, and Morris water maze tests. Compared to the CAA group, CAA mice treated with BDMC showed higher spontaneous alternation percentages in the Y‐maze, increased recognition indexes in novel object recognition, shorter latencies in the Morris water maze, more entries into the target quadrant, and a greater percentage of time spent in the target quadrant. These results indicate cognitive decline in CAA mice and suggest that BDMC treatment can improve these deficits. Furthermore, there were no significant differences in swimming speed among the three groups, indicating comparable motor abilities (Figure 3).

### 3.2 BDMC Reduces Aβ Burden in CAA Mice and Ameliorate Vascular Amyloid Deposition

**FIGURE 4:**
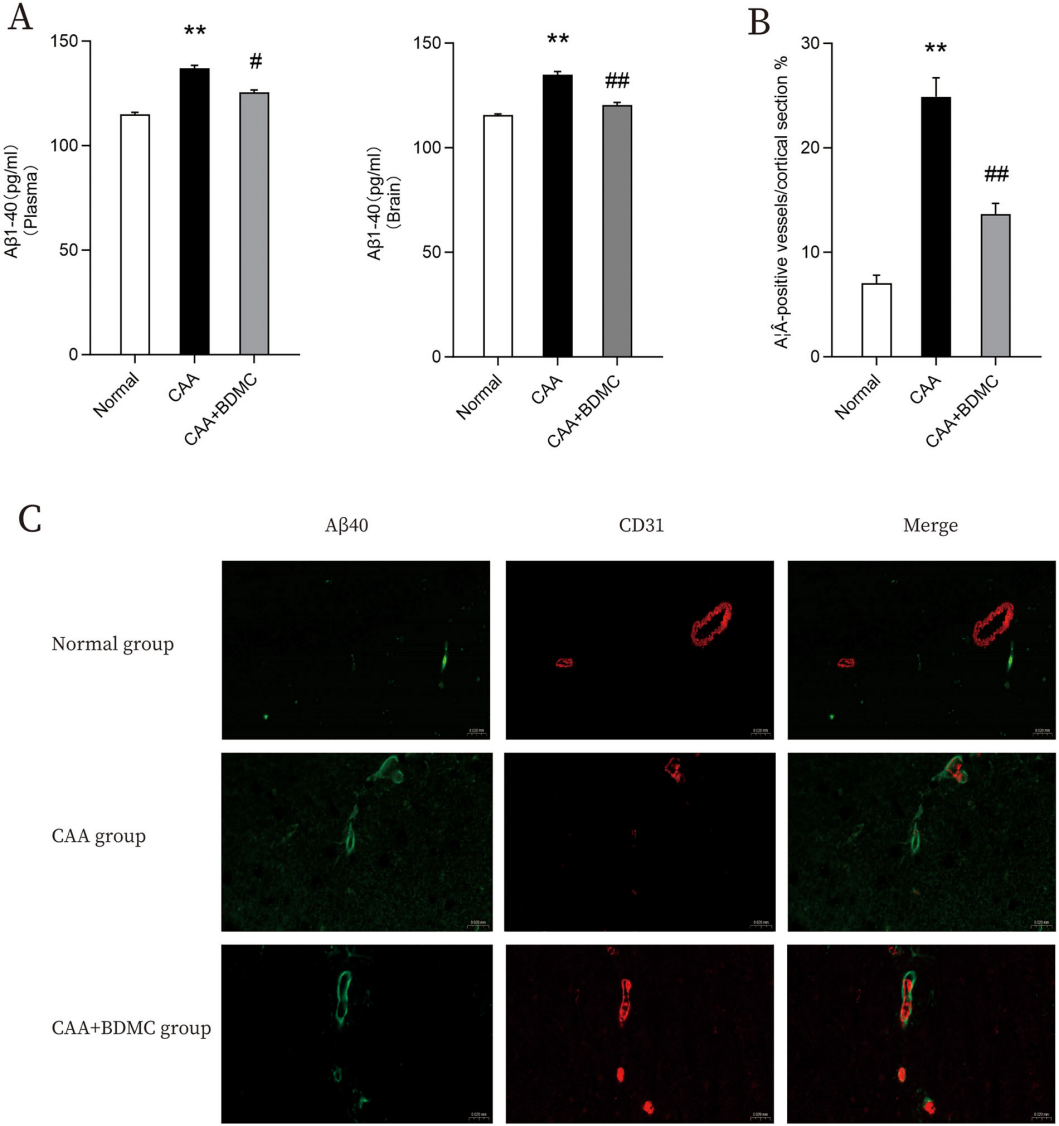
BDMC reduces Aβ burden in CAA mice and ameliorate vascular amyloid deposition. (A) Aβ40 levels in plasma and brain tissue homogenates. ELISA was performed to detect Aβ40 in plasma and brain homogenates. (B) Statistical graph depicting the number of Aβ40‐positive blood vessels. (C) Representative images of double immunofluorescence staining of small cerebral arteries in each group of mice. Scale bar: 20 µm; Aβ40 (green) co‐stained with endothelial cell marker CD31 (red). Data are shown as the mean ± SEM. Compared to the normal group: *p <0.05, **p <0.01; Compared to the CAA group: #*p* <0.05, ##*p* < 0.01.

Vascular amyloid deposition, a distinctive feature of CAA, differs from the typical presentation of Alzheimer's disease (AD). Despite both conditions involving the deposition of insoluble Aβ, AD patients mainly show significant plaque deposits rich in Aβ42 in the hippocampus and neocortex. In contrast, CAA patients commonly present vascular amyloid deposits, predominantly in the form of Aβ40, localized in the temporal and parietal cortical arteries, with fewer prominent amyloid plaque deposits. Comparative analysis with the normal group revealed a significant increase in Aβ40 content in the plasma and brain homogenate of mice in the CAA group. Immunofluorescence staining results demonstrated co‐localization of amyloid protein Aβ40 with the vascular endothelial cell marker CD31 in brain tissue, while amyloid plaques were infrequent, confirming the successful modeling of CAA mice. After a 2‐week BDMC treatment, a notable reduction in Aβ40 content was observed in the plasma and brain homogenate of CAA group mice. Concurrently, pathological changes in frozen brain tissue sections indicative of CAA decreased, suggesting that BDMC can mitigate Aβ load in CAA mice. These findings highlight the anti‐amyloid properties and therapeutic potential of BDMC in attenuating Aβ‐associated pathology (Figure 4).

### 3.3 BDMC Alleviates Microhemorrhages in CAA Mice

**FIGURE 5:**
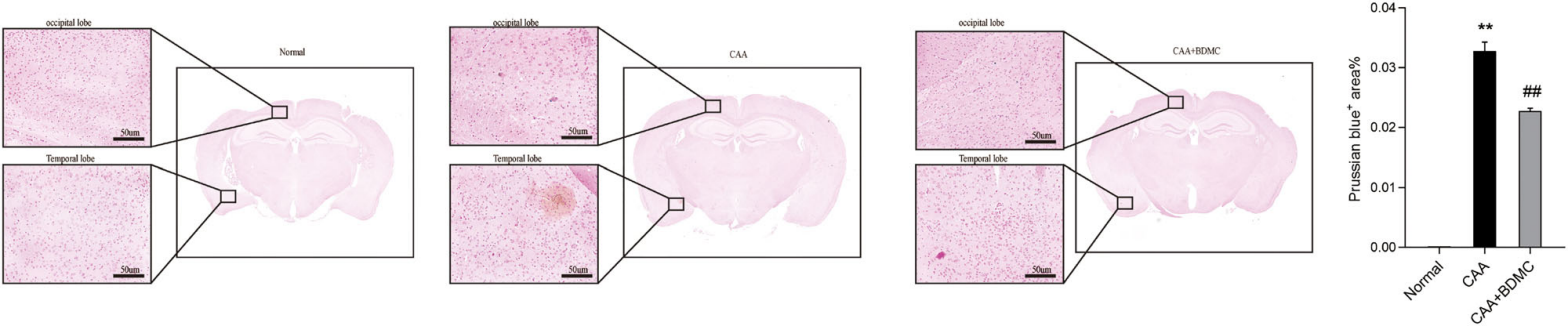
BDMC alleviates microhemorrhages in CAA mice. Results of Prussian blue staining on brain tissue slices of mice. Analyzed using Case Viewer software, scale bar: 50 µm. Blue dots represent Prussian blue‐positive staining, indicating deposition of hemosiderin. The number and area of blue dots correlate with the severity of microhemorrhages.

We conducted Prussian blue staining on mouse brain tissue slices to observe hemosiderin deposition. Compared to the control group, CAA group mice exhibited a significant increase in microhemorrhagic points in brain tissue. After BDMC treatment, there was a notable reduction in hemosiderin deposition in the brain tissue of CAA mice (Figure 5).

### 3.4 BDMC Reduces Expression of Proinflammatory Factors in CAA Mice

**FIGURE 6:**
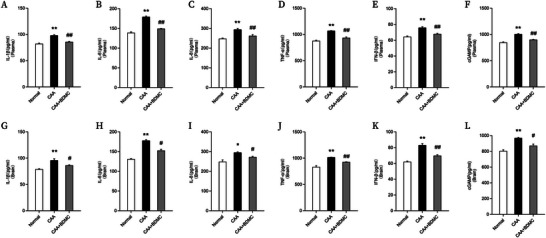
BDMC reduces expression of proinflammatory factors in CAA mice. (A–F) Statistical graphs showing levels of IL‐1β (A), IL‐6 (B), IL‐8 (C), TNF‐α (D), INF‐β (E), and cGAMP (F) in plasma of each group of mice. (G–L) Statistical graphs showing levels of IL‐1β (G), IL‐6 (H), IL‐8 (I), TNF‐α (J), INF‐β (K), and cGAMP (L) in brain homogenates of each group of mice. Data are shown as the mean ± SEM. Compared to the normal group: *p <0.05, **p <0.01; Compared to the CAA group: #*p* <0.05, ##*p* <0.01.

To assess the impact of BDMC on proinflammatory cytokine levels in CAA mice, we measured IL‐1β, IL‐6, IL‐8, TNF‐α, INF‐β, and cGAMP concentrations in both plasma and brain tissue using ELISA. ELISA results demonstrated a significant increase in proinflammatory cytokine expression in CAA mice, indicating heightened inflammatory response. However, after a 2‐week BDMC intervention, the CAA+BDMC group showed a significant reduction in IL‐1β, IL‐6, IL‐8, TNF‐α, INF‐β, and cGAMP concentrations in both plasma and brain tissue. This suggests that BDMC intervention effectively suppresses the elevated expression of proinflammatory factors in CAA mice (Figure 6).

### 3.5 BDMC Suppresses cGAS/STING Pathway Activation in CAA Mice

**FIGURE 7:**
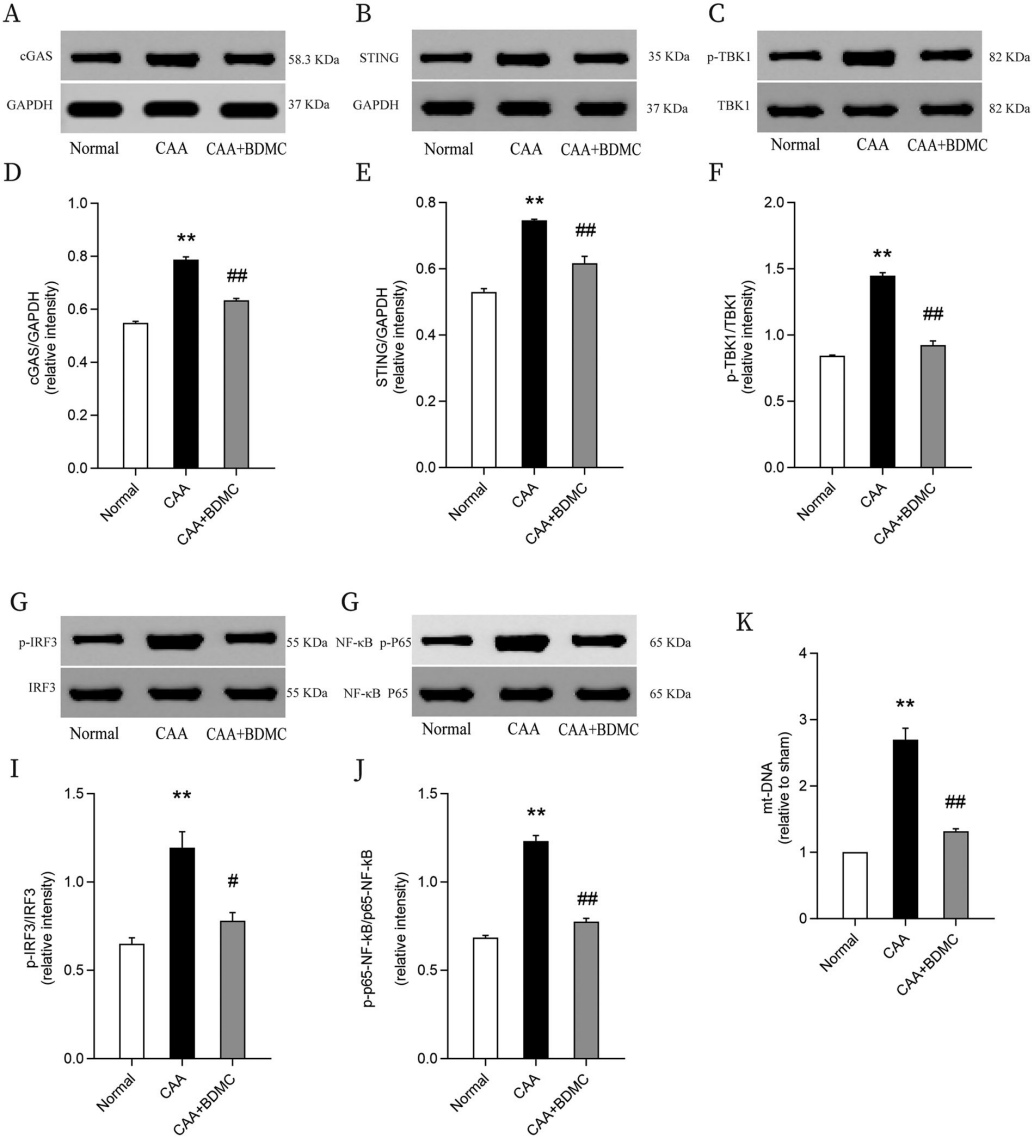
BDMC suppresses cGAS/STING pathway activation in CAA mice. (A–J) Representative and statistical graphs showing protein levels of cGAS, STING, TBK1, p‐TBK1, IRF3, p‐IRF3, p65‐NF‐κB, and p‐p65‐NF‐κB in brain tissue of each group of mice. (K) Statistical graph showing levels of mitochondrial DNA (mt‐DNA) in brain tissue of each group of mice. Data are shown as the mean ± SEM. Compared to the normal group: *p <0.05, **p <0.01; Compared to the CAA group: #*p* <0.05, ##*p* <0.01.

The cGAS/STING signaling pathway plays a crucial role in inflammatory responses in the central nervous system central nervous system inflammatory responses. To explore its involvement in the CAA model, we performed WB analysis to examine the expression of key pathway proteins, including cGAS, STING, TBK1, p‐TBK1, IRF3, p‐IRF3, p65‐NF‐κB, and p‐p65‐NF‐κB. We also used polymerase chain reaction (PCR) to measure mitochondrial DNA (mt‐DNA) content in brain tissues. Compared to the normal group, CAA mice exhibited increased levels of cGAS, STING, p‐TBK1, p‐IRF3, and p‐p65‐NF‐κB in brain tissues. However, the CAA+BDMC group showed significantly reduced expression of these cGAS/STING pathway‐related proteins compared to the CAA group. Additionally, while the levels of TBK1, IRF3, and p65‐NF‐κB did not change significantly, their phosphorylated forms were significantly increased in brain tissues. This suggests that the cGAS/STING pathway is activated in the CAA mouse model, and BDMC intervention can suppress this activation. PCR analysis of brain tissue mt‐DNA content supported this finding, further indicating the involvement of the cGAS/STING signaling pathway in the pathogenic mechanism of CAA (Figure 7).

### 3.6 BDMC Inhibits Necroptosis in CAA Mice

**FIGURE 8:**
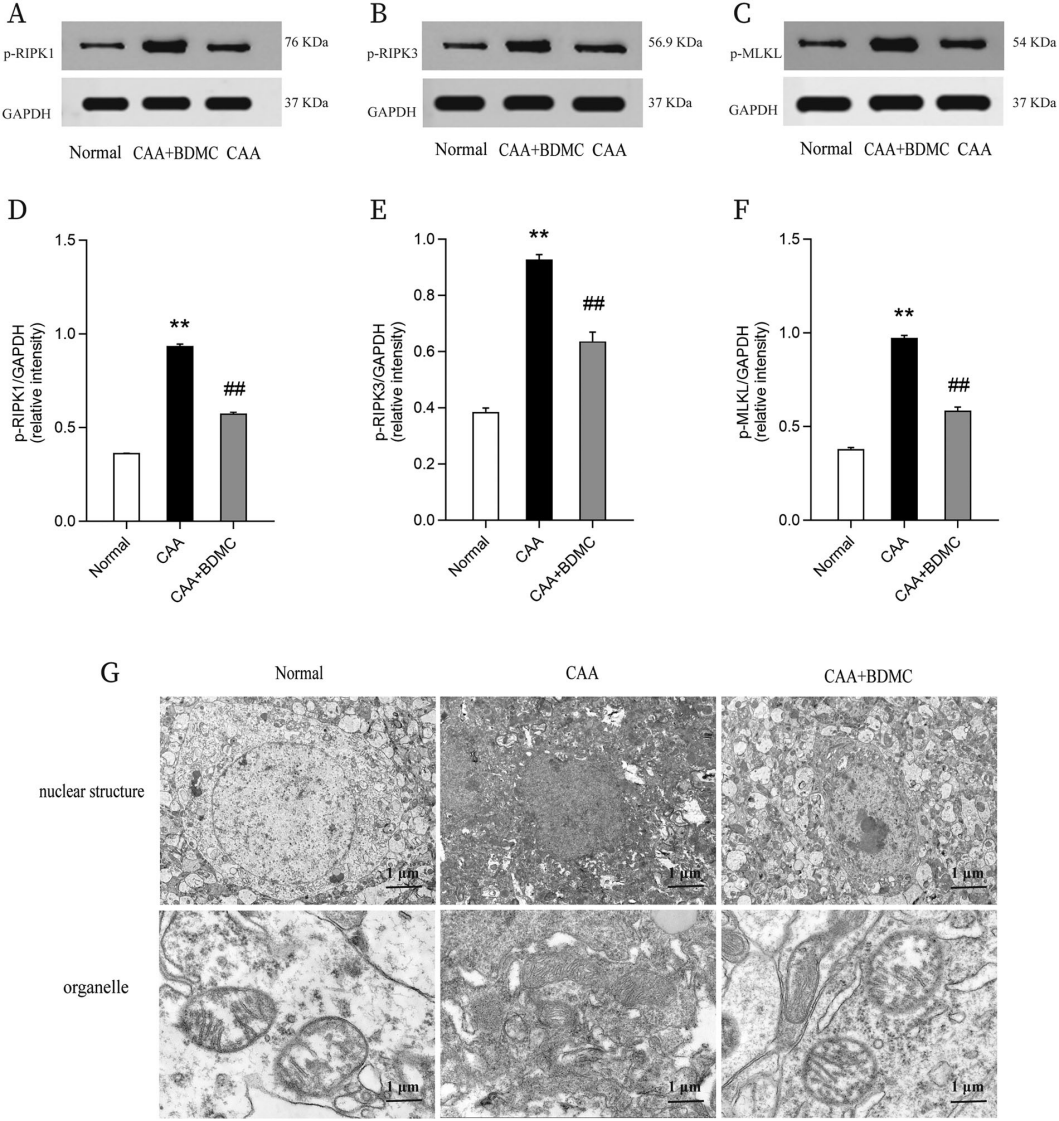
BDMC inhibits necroptosis in CAA mice. (A–F) Statistical graphs showing expression levels of key proteins in the necroptosis pathway (p‐RIPK1, p‐RIPK3, p‐MLKL) in brain tissue of mice. (G) Transmission electron microscopy images of mouse brain tissue. Upper panel: Morphology of nuclear structures under electron microscopy. Lower panel: Morphology of organelles under electron microscopy. Data are shown as the mean ± SEM. Compared to the normal group: *p <0.05, **p <0.01; Compared to the CAA group: #*p* <0.05, ##*p* <0.01. Graphical Working model indicates the effects of BDMC on cerebral amyloid angiopathy mouse model. BDMC exerts therapeutic effects in a mouse model of CAA established through chronic treatment involving five vascular risk factors, it inhibits neuroinflammatory responses by suppressing the expression of cGAS/STING signaling pathway proteins and reduces ameliorate vascular amyloid deposition.

To investigate necroptosis levels and the impact of BDMC treatment in CAA mice, we conducted Western blot (WB) analysis to assess the protein levels of p‐RIPK‐1, p‐RIPK‐3, and p‐MLKL in mouse brain tissues. Additionally, we used transmission electron microscopy to observe neuronal cell morphology. WB results showed that CAA mice exhibited increased necroptosis levels along with nuclear condensation, cell membrane rupture, cellular and organelle swelling, and significant chromatin fragmentation, indicating the presence of necroptosis in the murine model of cerebral amyloid angiopathy. However, the CAA+BDMC group demonstrated lower protein levels of p‐RIPK‐1, p‐RIPK‐3, and p‐MLKL in brain tissues compared to the CAA group, as observed through WB analysis. Furthermore, the CAA+BDMC group showed improved pathological features associated with necroptotic cell death in neuronal cells. These findings suggest that BDMC treatment effectively inhibits necroptosis in CAA mice, highlighting its potential therapeutic impact on cerebral amyloid angiopathy (Figure 8).

## 4 Discussion

Cerebral amyloid angiopathy (CAA) is an age‐related disease, with 50% of elderly individuals exhibiting pathological changes associated with CAA (Charidimou et al. 2017; Owasil et al. 2020; Jäkel et al. 2021). Clinical interventions mainly target acute or chronic cerebrovascular events or dementia stemming from CAA, yet their effectiveness remains uncertain, leading to an overall poor prognosis (Kozberg et al. 2020; Inoue et al. 2021). The lack of mature CAA animal models further constrains research on the pathogenesis and treatment of CAA. Our exploration into such models has revealed that natural animal models like crab‐eating macaques, transgenic rats, and mouse CAA models—owing to their high costs, technical challenges, and high failure rates—have not seen widespread adoption, even no animal models currently represent the sporadic CAA pathologies (Jäkel et al. 2017).

The common sporadic form of CAA arises from a complex interplay of environmental, lifestyle, and vascular risk factors. Increasing evidence suggests that long‐term high‐fat diets, diabetes, stress, and vascular risk factors not only induce structural and functional changes in blood vessels (Smith 2017) but also increase the risk of dementia (Ng et al. 2010; Venkat, Chopp, and Chen 2015). Moreover, these factors are closely linked to inflammatory responses in both the peripheral and central nervous systems (Boitard et al. 2014; Wang et al. 2020). Studies have also indicated a significant relationship between copper and cognitive impairment, Crooks et al. (2020) discovered a strong co‐localization of copper ions with vascular amyloid deposits in the brains of individuals with sporadic CAA and familial Iowa‐type CAA, highlighting copper ions as structural cofactors promoting vascular amyloid formation. Bettina M Foidl, by inducing inflammatory reactions through intraperitoneal injection of lipopolysaccharide, streptozotocin‐induced diabetes, increased social stress via cage replacement, high‐fat diets, and copper supplementation in drinking water—representing five vascular risk factors—successfully established a CAA mouse model in normal C57BL/6 mice. After 35 weeks of treatment, typical vascular amyloid deposits were observed in brain tissues, although no deposits of β‐amyloid plaques and tau pathology were noted in the brain (Foidl and Humpel 2019). Given the pivotal role of vascular mechanisms in CAA onset, we adopted a similar approach to establish a CAA mouse model and conducted assessments, revealing a significant accumulation of vascular amyloid deposits in the brain tissues of mice exposed to chronic stimulation by multiple vascular risk factors, consistent with prior research findings.

The current consensus suggests that vascular deposition of Aβ plays a critical role in the pathogenesis of CAA (Bourassa et al. 2019). In patients with CAA, Aβ deposition within blood vessels may trigger vascular inflammatory responses (Solesio et al. 2018; Riley and Tait 2020), promoting the extravasation of immune cells, which in turn leads to widespread neuroinflammation and neurodegeneration (Parodi‐Rullán, Javadov, and Fossati 2021). In animal models of amyloidosis, such as Tg2576 and 3xTg mice, elevated levels of interleukin‐6 (IL‐6), tumor necrosis factor‐alpha (TNFα), and interleukin‐1β (IL‐1β) have been detected in cerebrospinal fluid (CSF) and plasma of CAA patients (Tripathy et al. 2013; Low et al. 2019). The levels of inflammatory responses have been demonstrated to positively correlate with Aβ levels in the brains of amyloidosis mouse models (Tesseur et al. 2006), underscoring the importance of inflammation in CAA pathogenesis. Previous research, including our own, has consistently demonstrated a significant increase in these inflammatory markers in both CAA patients and animal models.

The cGAS/STING pathway represents a crucial avenue in the inflammatory responses of the central nervous system (Decout et al. 2021), implicated in the pathogenesis of various neurodegenerative diseases, including Alzheimer's disease, Parkinson's disease, and amyotrophic lateral sclerosis (Glass et al. 2010). However, its role in cerebral vascular inflammation and amyloidosis remains to be definitively established. Due to the mediation of vascular inflammatory responses by Aβ, mtDNA is released into the cytoplasm following the disruption of endothelial cell mitochondria (Guo et al. 2020). The cyclic GMP‐AMP synthase (cGAS) is activated by mtDNA in a sequence‐independent manner, leading to the upregulation of cGAS/STING pathway‐related protein expression. This cascade further promotes the expression of proinflammatory cytokines and chemokines such as TNF‐α, IL‐1β, IL‐6, IL‐8, and INF‐β (Mcwhirter et al. 2009; Ablasser and Chen 2019). These proinflammatory cytokines infiltrate the brain parenchyma through the compromised blood‐brain barrier, resulting in the activation of astrocytes and microglial cells. All of these processes are associated with driving the neuroinflammatory response in the central nervous system. While there is currently no conclusive evidence demonstrating the regulation of the cGAS/STING pathway to mitigate inflammatory responses by BDMC, recent experiments indicate that bisdemethoxycurcumin may attenuate alcohol‐induced neuroinflammation by inhibiting the TLR4/NF‐κB signaling pathway (Mahattanadul et al. 2009). It may also expedite the healing of gastric ulcers by directly inhibiting inflammation mediated by inducible nitric oxide synthase (iNOS) (Cuesta et al. 2021). Additionally, BDMC has been observed to reduce glial cell activation, exerting neuroprotective effects, as validated in a rodent model of radiation‐induced injury (Xie et al. 2023). Therefore, we hypothesize that the cGAS/STING pathway may be involved in the neuroinflammatory mechanisms of CAA mice, and BDMC may regulate the cGAS/STING pathway to alleviate inflammatory responses. To explore this hypothesis, we utilized WB to assess the expression of cGAS/STING pathway‐related proteins and employed PCR to quantify mtDNA levels in brain tissues. The results aligned with our conjecture. However, it is regrettable that we did not investigate the use of agonists or antagonists, along with genetic manipulations such as knockdown or overexpression of relevant genes, to further validate and explore the regulatory mechanisms of the cGAS/STING pathway. This aspect awaits further in‐depth investigation in our future research endeavors.

Necroptosis is a form of necrotic cell death that occurs downstream of RIPK1 and RIPK3 activation and disruption of the plasma membrane by the pseudokinase MLKL (Cai et al. 2013). A recent study found that necroptosis requires STING‐dependent production of both IFN and TNF, highlighting a potential relationship between STING and necroptosis (Hu et al. 2022). Furthermore, this form of cell death can trigger age‐related neuroinflammation, impairing neuronal connections in the hippocampus and cognitive functions (Thadathil et al. 2021). In our study, we observed that BDMC alleviates necrotic apoptosis, a phenomenon possibly associated with the inhibition of the cGAS/STING pathway. Based on these observations, we propose that BDMC could be an effective treatment for age‐related cognitive decline and memory impairments.

In conclusion, treatment with BDMC significantly reduced the levels of Aβ40, IL‐1β, IL‐6, IL‐8, TNF‐α, INF‐β, and cGAMP in both the brain tissue and plasma of CAA mice. This treatment also decreased the deposition of Aβ in cerebral blood vessels and the presence of iron‐containing hemosiderin deposits. Additionally, there was a reduction in the brain tissue levels of cGAS, STING, p‐TBK1, p‐IRF3, p‐p65‐NF‐κB, mtDNA, p‐RIPK‐1, p‐RIPK‐3, and p‐MLKL. Improvements were noted in neuronal cell nuclear condensation, membrane rupture, swelling of the cell body and organelles, and chromatin fragmentation. Furthermore, CAA mice treated with BDMC demonstrated enhanced cognitive and spatial memory, evidenced by increased spontaneous alternation in the Y‐maze, a higher recognition index in novel object recognition tests, shorter latency periods in the Morris water maze, more frequent entries into the target zone, and longer durations spent in the target zone. Our study establishes a theoretical and experimental basis for BDMC as a promising therapeutic candidate for the treatment of CAA.

Definitely, our study has several limitations. First of all we unable to pinpoint, which single stimulus drives, forces and triggers the progression of the disease. The exact cellular and molecular mechanisms linking the onset of the disease to these vascular risk factors remain unclear. Additionally, we did not investigate peripheral vasculature after treatment with the five vascular risk factors. Lastly, we did not inhibit cGAS/STING signaling to determine whether BDMC exerts its effects via this pathway. Thus, further studies are needed to evaluate the treatment effects of BMDC in mice with CAA and the underlying mechanism related to cGAS/STING signaling.

## Author Contributions

**Shudong Lin**: Methodology, investigation. **Guanghua Zhu**: Methodology, investigation. **Juan Xie**: Methodology, investigation. **Xuanwei Wen**: Methodology. **Limin Deng**: Methodology. **Sijing Li**: Methodology. **Guozhi Liu**: Methodology. **Feiyan Wang**: Methodology. **Shuangxi Chen**: Investigation, writing–original draft. **Zijian Xiao**: Funding acquisition, writing–review and editing, writing–original draft, conceptualization.

## Conflicts of Interest

The authors declare no conflicts of interest.

### Peer Review

The peer review history for this article is available at https://publons.com/publon/10.1002/brb3.70245.

## Data Availability

The data that support the findings of this study are available from the corresponding author upon reasonable request.
